# Mechanisms of ROS Regulation of Plant Development and Stress Responses

**DOI:** 10.3389/fpls.2019.00800

**Published:** 2019-06-25

**Authors:** Honglin Huang, Farhan Ullah, Dao-Xiu Zhou, Ming Yi, Yu Zhao

**Affiliations:** ^1^National Key Laboratory of Crop Genetic Improvement, Huazhong Agricultural University, Wuhan, China; ^2^College of Science, Huazhong Agricultural University, Wuhan, China

**Keywords:** ROS, plant development, stress response, epigenetic modification, regulatory mechanism

## Abstract

Plants are subjected to various environmental stresses throughout their life cycle. Reactive oxygen species (ROS) play important roles in maintaining normal plant growth, and improving their tolerance to stress. This review describes the production and removal of ROS in plants, summarizes recent progress in understanding the role of ROS during plant vegetative apical meristem development, organogenesis, and abiotic stress responses, and some novel findings in recent years are discussed. More importantly, interplay between ROS and epigenetic modifications in regulating gene expression is specifically discussed. To summarize, plants integrate ROS with genetic, epigenetic, hormones and external signals to promote development and environmental adaptation.

## Introduction

It is well known that improving crop yield and productivity requires improved understanding of the coordinated growth of plant tissues and organs. Plant morphogenesis is regulated by both intrinsic genetic programmes and external environmental factors. Reactive oxygen species (ROS) are regarded as by-products of plant aerobic metabolism and are generated in several cellular compartments such as chloroplasts (Dietz et al., 2016), mitochondria (Huang et al., 2016), and peroxisomes (Sandalio and Romero-Puertas, 2015). ROS not only cause irreversible DNA damage and cell death, but also function as important signaling molecules that regulate normal plant growth, and responses to stress. This suggests that ROS have a dual role *in vivo* depending on different levels of reactivity, sites of production and potential to cross biological membranes (Miller G. et al., 2010). From an evolutionary point of view, the emergence of oxygen-releasing photosynthetic life had a profound impact on all living organisms (Rosing and Frei, 2004). As the source of all ROS, oxygen (O_2_) is stable and not very reactive in plants. However, it can be converted into high-energy ROS in several organelles by various processes that affect plant metabolism (Mittler, 2017). As reactive molecules, ROS oxidize and modify some cellular components and prevent them from performing their original functions (Apel and Hirt, 2004; Mittler et al., 2004). Under unfavorable circumstances, plants generate a large number of ROS species involved in regulation of various processes including pathogen defense, programmed cell death (PCD), and stomatal behavior (Gill and Tuteja, 2010; Schippers et al., 2016). These reactions exert profound or irreversible effects on development of tissues and organs, often leading to abnormal plant growth or death (Mittler, 2017; Tognetti et al., 2017). Additionally, ROS interplay with epigenetic modifiers and hormones to control plant developmental processes, and stress responses (Gill and Tuteja, 2010; Tsukagoshi et al., 2010; Zeng et al., 2017; Kong et al., 2018). In general, low ROS levels are necessary for the progression of several basic biological processes, including cellular proliferation and differentiation (Tsukagoshi et al., 2010; Zafra et al., 2010). At higher levels ROS pose a significant threat that may eventually lead to DNA damage, and incorrect timing of PCD directly (Xie et al., 2014).

## Generation and Removal of ROS in Plants

In plants, ROS exist in ionic and/or molecular states. Ionic states include hydroxyl radicals (^•^OH) and superoxide anions ($O_{2}^{\cdot -}$), while molecular states mainly include hydrogen peroxide (H_2_O_2_), and singlet oxygen (^1^O_2_) (Blokhina, 2003; Apel and Hirt, 2004; Mittler et al., 2004). Each type of ROS has a different oxidative capacity and affects different physiological and biochemical reactions regulated by different genes in plants. As an excited oxygen, singlet oxygen (^1^O_2_) is usually generated in chloroplast photosystem II (PSII) and has strong oxidizability. Although ^1^O_2_ exists for a very short time and is extremely unstable in cells, once generated, it has great impact on photosynthesis. Superoxide anion ($O_{2}^{\cdot -}$) is the precursor of various ROS because of its instability and strong oxidation/reducibility. $O_{2}^{\cdot -}$ could maintain the stability of plant stem cells (Zeng et al., 2017). However, excessive $O_{2}^{\cdot -}$ also causes increased ROS levels and eventually leads to cell death (Gill and Tuteja, 2010). In rice, roots, and stems seem to be the main organs of $O_{2}^{\cdot -}$ production, which might be related to their adaptation to the aquatic environment (Yamauchi et al., 2017). $O_{2}^{\cdot -}$ can be produced by photosynthetic electron transport chains, mitochondrial respiratory electron transport chains, and membrane-dependent NADPH oxidase (RESPIRATORY BURST OXIDASE HOMOLOG proteins) systems, which react with hydrogen ions to form oxygen molecules or with superoxide dismutase (SOD) to form H_2_O_2_ (Bose et al., 2014; Mhamdi and Van Breusegem, 2018). Among these, H_2_O_2_ is considered an important redox molecule, given its specific physical and chemical properties, including a remarkable stability within cells (half life of 10^–3^ s), and rapid and reversible oxidation of target proteins (Mittler, 2017; Mhamdi and Van Breusegem, 2018). H_2_O_2_ can be transported by aquaporins localized in the cell membrane, not only causing long-distance oxidative damage (Bienert et al., 2007; Wudick et al., 2015), but also participating in cell signaling regulation (Miller E.W. et al., 2010). H_2_O_2_ has been shown to participate in cell differentiation, senescence, PCD, and cell wall formation in plants (Moller et al., 2007; Karkonen and Kuchitsu, 2015; Schippers et al., 2016; Waszczak et al., 2016; Ribeiro et al., 2017; Zeng et al., 2017). Additionally, H_2_O_2_ interplays with hormones to regulate plant developmental process and stress responses. ^•^OH can be formed when the O−O double bond in H_2_O_2_ cleaves. ^•^OH is active and usually acts very near its production site. Therefore, ^•^OH is the most reactive ROS, and it can react with all biological molecules. It can oxidize the cell wall polysaccharides, resulting in cell wall loosening (Karkonen and Kuchitsu, 2015), and it can also induce DNA single-strand breakage (Hiramoto et al., 1996). Under normal conditions, excessive ROS can be scavenged by various antioxidative defense mechanisms. The equilibrium between production and scavenging of ROS may be perturbed by various biotic and abiotic stresses. These disturbances of the equilibrium can cause sudden increases in intracellular ROS levels and significantly damage cell structures. Taken together, plants are obliged to cope with excessive ROS generation in order to maintain cellular redox homeostasis. Accordingly, the augmented ROS levels are sensed and restrictively controlled by a battery of ROS-scavenging systems.

ROS scavenging mechanisms can be classified into two types: enzymatic and non-enzymatic antioxidant defense systems, which work synergistically and interactively to neutralize free radicals. The enzymatic systems mainly include SOD, catalase (CAT), ascorbate peroxidase (APX) and glutathione peroxidase (GPX) (Apel and Hirt, 2004). In rice, most of these genes participating in ROS removal exhibit tissue/organ-specific expression profiles (Table 1). However, their function in ROS homeostasis and regulation of gene expression remain unclear. Among the enzymatic systems, SOD is able to rapidly convert ⋅OH to H_2_O_2_, and the generated H_2_O_2_ is then converted to water and dioxygen by peroxidase and CAT (Gechev et al., 2006; Mittler, 2017). The non-enzymatic systems are mainly mediated by low molecular mass antioxidants, such as glutathione, ascorbic acid (AsA) and flavonoids, which are known to remove hydroxyl radicals and singlet oxygen (Gechev et al., 2006). When ROS levels in the cell exceed the range of the scavenging systems, cells enter the oxidative state, resulting in oxidative modification and cell damage which can lead to death. When ROS levels are low, the cells are in the reduced state, and ROS can be used as second messengers that participate in stem cell maintenance, cell division, and differentiation, organogenesis, and biotic and abiotic responses, etc. (Fujita et al., 2006; Zeng et al., 2017). Thus, it is necessary to maintain ROS levels within the right range for plant health. Of course, alterations in ROS levels that are part of the normal function of the plant should not exceed the threshold boundary between cytostatic and cytotoxic levels.

**TABLE 1:** Genes that participate in the removal of ROS and their expression profiles in various rice tissues and organs.

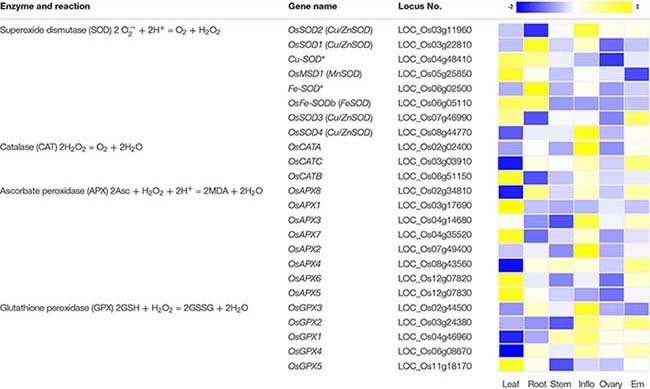

*Genes information from the Rice Annotation Project (RAP); heatmap data from the Rice cpssnmExpression Profile Databasecpesnm (RiceXPro, http://ricexpro.dna.affrc.go.jp/). Inflo, inflorescence; Em, Embryo.*

## Roles of ROS in Plant Growth and Development

The appearance of aerobic conditions gave organisms the opportunity to use oxygen as an electron acceptor, while enabling them to harness its reactive properties for metabolism and signaling (Schippers et al., 2012; Foyer and Noctor, 2016). It was therefore, inevitable that evolution in an oxygenic environment would necessitate integration of oxidative processes and ROS sensing and signaling into the developmental programs. From seed germination to plant senescence, ROS are dynamically generated or removed, which makes plants regulate their development in order to adapt to different environments. However, the effects of ROS on plant growth and development are more complex due to the temporal and spatial variability of ROS regeneration and interplay between them in plants.

### ROS Participate in the Maintenance of Plant Vegetative Apical Meristems

**FIGURE 1:**
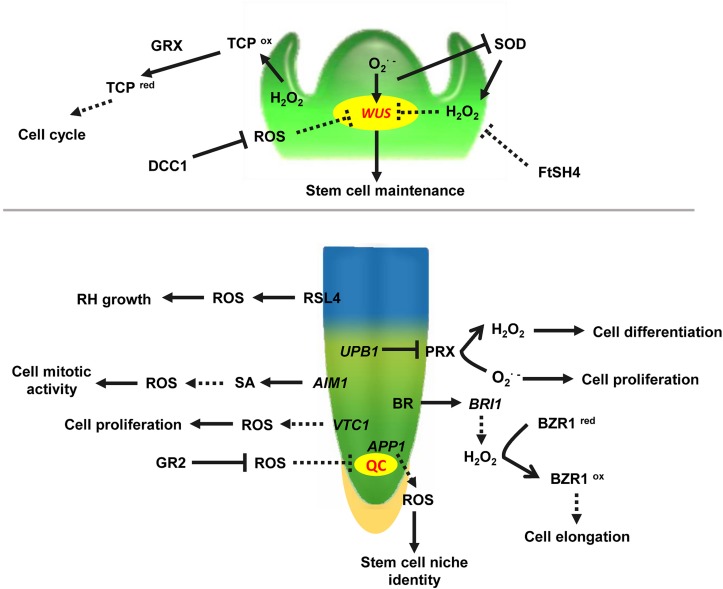
ROS involved in vegetative apical meristem identity in the shoot apical meristem. WUS, WUSCHEL, a regulator maintaining stem cell identity in shoot apical meristem. $O_{2}^{\cdot -}$ and H_2_O_2_ activate and repress WUS activity to balance stem cell identity and differentiation, respectively. DCC1, a functional thioredoxin that inhibits the accumulation of ROS in the cell, and creates conditions for SAM formation. TCP, TEOSINTE BRANCHED/CYCLOIDEA/PCF, a transcriptional regulator of the cell cycle, is inhibited by higher ROS levels in the SAM. TCP^ox^ (TCP in oxidated state)/TCP^red^ (TCP in reduced state) can be regulated by GRX (Glutaredoxin). FtSH4 (also named AtFTSH4), an ATP-dependent mitochondrial protease, associated with internal oxidative stress and mitochondrial function in the SAM. UPB1, transcription factor UPBEAT1, plays an important role in maintaining $O_{2}^{\cdot -}$ and H_2_O_2_ balance in the RAM. RSL4, ROOT HAIR DEFECTIVE SIXLIKE 4, a bHLH transcription factor, activates ROS-related gene expression and regulates root hair elongation. *AIM1*, *ABNORMAL INFLORESCENCE MERISTEM*, regulates ROS levels via the SA synthesis pathway in the RAM. *APP1*, *Arabidopsis thaliana P-loop NTPase1*, controls ROS homeostasis in the stem cell niche (SCN) of the root tip. VTC1, an Enzyme Involved in Ascorbate Biosynthesis, regulates H_2_O_2_ levels in the RAM. BRI1, BRASSINOSTEROID INSENSITIVE 1, and BZR1, BRASSINAZOLE-RESISTANT1, are modified by ROS. QC, quiescent center; BR, brassinosteroids; SA, salicylic acid; GR, glutathione reductase; RH, root hair. Arrows indicate positive regulation. Bars indicate negative regulation. Unbroken lines indicate direct regulation, and broken lines indicate unclear mechanism.

Emerging evidence indicates that ROS homeostasis shapes plant vegetative apex development (Figure 1). In *Arabidopsis thaliana*, $O_{2}^{\cdot -}$ primarily accumulates in the meristematic zone of the root tip and is required for cell division, whereas H_2_O_2_ mainly accumulates in the elongation zone, which confers cell differentiation (Tsukagoshi et al., 2010). These two different ROS micro-environments coincide with the meristematic and the elongation zone, and their distributions are important for localization of the transition zone. Because of the ROS species gradients, cells entering the transition zone can still proliferate. Once the ratio of $O_{2}^{\cdot -}$ to H_2_O_2_ reaches a certain level, cells stop dividing and begin to elongate (Dunand et al., 2007). Therefore, the ROS balance in the transition zone is essential. The transcription factor UPB1 (UPBEAT1) is one of the key regulators maintaining this balance. Further studies showed that H_2_O_2_ itself affects *UPB1* expression, and this regulatory system contains a feedback loop that plays a role in both ROS homeostasis and root growth (Tsukagoshi et al., 2010). In addition, the quiescent center (QC) and distal stem cell (DSC) are required for root apical meristem (RAM) size maintenance. *APP1* (*Arabidopsis thaliana P-loop NTPase1*) affects root stem cell niche (SCN) identity through its control of local ROS homeostasis. Disruption of *APP1* is accompanied by a reduction in ROS levels, a rise in the rate of cell division in the QC, and the promotion of root DSC differentiation, suggesting that ROS levels are directly related to RAM size in *Arabidopsis* (Yu et al., 2016). More importantly, ROS, together with hormones and other signal molecules, regulate plant root primary growth. In the RAM, ROS, and auxin signaling are antagonistically regulated to balance root meristem growth (Tognetti et al., 2017). The imbalance of different ROS species or accumulation of ROS induced by high levels of glucose oxidizes active IAA, resulting in its degradation, impairs root meristem activity, and subsequently inhibits root growth through the conserved macroautophagy/autophagy pathway (Huang et al., 2019). The findings suggest that autophagy is an essential mechanism for glucose-mediated maintenance of the root meristem by modulating the homeostasis of cellular ROS and promoting the degradation of the oxidatively damaged peroxisomes. Recent studies have shown that Brassinosteroids (BRs) also control root tip stem cell activity through ROS. Binding of BR to receptor kinase BRI1 (BRASSINOSTEROID INSENSITIVE1) increases cellular levels of H_2_O_2_, and the increased H_2_O_2_ induces oxidative modification of BZR1 (BRASSINAZOLE-RESISTANT1) and BES1 (BRI1-EMSSUPPSSOR1), the key transcription factors in BR signaling. The oxidative modification enhances BZR1 transcriptional activity by promoting its interaction with PIF4 (PHYTOCHROME INTERACTING FACTOR4) and ARF6 (AUXIN RESPONSE FACTOR6), thereby promoting root meristem development (Lv et al., 2018; Tian et al., 2018). However, at present, there is limited information about the relationship between ROS and cytokinins in regulation of apex growth. Taken together, it is clear that the hormonal and ROS networks can no longer be regarded as independent mechanisms. Rather, they are interconnected in order to trigger physiological and stress adaptation responses. Glutathione reductase (GR) plays a key role in controlling the levels of reduced glutathione in the *Arabidopsis* RAM. Excessive accumulation of oxidized glutathione in *GR2* (*Glutathione reductase2*) mutants results in root apical cells entering the oxidized state and eventually leads to abnormal growth. Exogenous application of reduced glutathione partially restores the normal root phenotype. Glutathione regulates ROS levels in cells through both auxin/PLETHORA (PLT) dependent and independent pathways, thereby participating in and maintaining ROS homeostasis in the RAM (Yu et al., 2013). *VTC1*, which is a rate-limiting gene affecting the quantity of AsA, as well as several downstream steps, is shown to be a key modulator of H_2_O_2_ levels. Knockout of *VTC1* resulted in elevated H_2_O_2_ levels and numbers of QC cells and periclinal divisions in the RAM (Kka et al., 2018). This result revealed the interaction between AsA and H_2_O_2_ in maintaining RAM size. Accumulating evidence has shown that the redox state of the cell affects its proliferation/differentiation program. For example, in *Arabidopsis* embryonic roots, division of cells in G1 phase is accelerated if they are in the oxidized state and slowed if they are in the reduced state (de Simone et al., 2017). These results suggest that controlled oxidation is a key feature of the early stages of the plant cell cycle.

In the *Arabidopsis* shoot apical meristem (SAM), enrichment of $O_{2}^{\cdot -}$ in stem cells activates the *WUSCHEL* gene to maintain stem cell activities, whereas H_2_O_2_ accumulation in the peripheral zone (PZ) promotes cell differentiation. Moreover, H_2_O_2_ negatively regulates $O_{2}^{\cdot -}$ generation in stem cells, and increased H_2_O_2_ levels or $O_{2}^{\cdot -}$ scavenging leads to the termination of stem cells (Zeng et al., 2017). These results suggest that ROS mediate the control of plant stem cell fate, and the balance between $O_{2}^{\cdot -}$ and H_2_O_2_ is essential for shoot stem cell maintenance and differentiation. The expression of transcription factor TCP (TEOSINTE BRANCHED/CYCLOIDEA/PCF), which is associated with the cell cycle, is inhibited by higher ROS levels in the SAM. At the same time, glutaredoxin (GRX) reduces ROS levels in the SAM. Lower ROS levels activate *TCP* and directly regulate the expression of cell cycle-related genes *CYCA2;3*, and *CYCB1;1*, thus promoting SAM cell division and maintaining SAM stability (Viola et al., 2013; Schippers et al., 2016). Recently, a novel thioredoxin DCC1 has been shown to determine the shoot regeneration capacity of various *Arabidopsis* ecotypes. Further studies demonstrated that DCC1 altered the activity of respiratory chain NAD(P)H dehydrogenase complex I. Knock-out of *DCC1* triggered production of mitochondrial ROS. The process further regulates shoot regeneration. Meanwhile, six different SNPs (Single Nucleotide Polymorphism) in the *DCC1*gene sequence were found to be closely related to bud regeneration in different *Arabidopsis* ecotypes, and ROS levels varied in ecotypes harboring different SNPs (Zhang et al., 2018). Taken together, these results demonstrate that modulation of ROS homeostasis plays an essential role in many processes from apical meristem maintenance to *de novo* shoot initiation.

### ROS Trigger Plant Organ Morphogenesis

As signaling components, ROS are distributed in all plant tissues, especially in metabolically active tissues. Maintenance of ROS homeostasis and ROS generation regulates seed germination through GA and/or ABA metabolism and signaling in *Arabidopsis* and barley, respectively (Baek et al., 2015; Ishibashi et al., 2015). Late embryogenesis abundant protein OsLEA5 interacted with zinc finger transcription factor ZFP36 to co-regulate ABA-inhibited seed germination by controlling the expression of APX *OsAPX1* in rice (Huang et al., 2018). Phenylalanine (Phe) biosynthetic activity of AROGENATE DEHYDRATASE3 (ADT3) played a critical role in coordinating ROS homeostasis and cotyledon development in etiolated *Arabidopsis* seedlings. The results revealed that cytosolic Phe played a critical role during the transition of seedlings from heterotrophy to autotrophy by protecting the cells from oxidative damage, and by providing substrates for defense (Para et al., 2016). ROS are also thought to play essential roles in leaf development, senescence and organ dormancy. For instance, expression of *CAT2* (*Catalase 2*) is reduced in the leaves of *Arabidopsis* upon bolting. This results in accumulation of H_2_O_2_ which promotes the expression of the *WRKY53* gene, which is required for leaf senescence (Zimmermann et al., 2006). Loss of function of mitochondrial ATP-dependent protease FtSH4 increases ROS, and is involved in leaf senescence via regulation of WRKY-dependent salicylic acid (SA) accumulation and signaling (Zhang et al., 2017c). Potato tuber dormancy is a complicated physiological process. When $O_{2}^{\cdot -}$ levels are inhibited, tuber sprouting is delayed. When treated with exogenous H_2_O_2_, potato tuber dormancy is released (Liu et al., 2017). This indicates that different ROS play different roles during the progression of potato tuber dormancy. Similarly, the concentration of H_2_O_2_ also dynamically changes in olives as they progress from bud formation through fertilization, which indicates that the difference in H_2_O_2_ concentrations has important physiological significance for the development of different organs (Zafra et al., 2010). During the process of *Arabidopsis* vernalization and flowering, the content of ROS initially increases and then decreases. This suggests that from the initiation of floral buds to maturation of sexual organs, ROS levels might play different roles (Zimmermann et al., 2006). In rice, homeobox gene *MADS3* has been proved to be essential for stamen formation during early floral development. However, at later stages of anther development, *MADS3* regulates ROS homeostasis, and abnormal expression of *MADS3* causes the accumulation of $O_{2}^{\cdot -}$ and pollen sterility (Hu et al., 2011). PEROXIDASE9 and PEROXIDASE40, which catalyze the oxidation of various substrates by H_2_O_2_, are genetically redundant and essential for proper anther and pollen development in *Arabidopsis*, likely through their extensin cross-linking activity (Jacobowitz et al., 2019). OsCIPK31 perceives the response to stresses and regulates ROS accumulation and IAA distribution in the panicle. Excess IAA might lead to ROS accumulation in the apical spikelet, which ultimately leads to cell death in rice panicles (Peng et al., 2018). H_2_O_2_ and superoxide are formed during lateral root (LR) development, and contribute to the elongation of LRs but, intriguingly, not to the initiation of LR primordia (Manzano et al., 2014). In addition, ROS are also essential for the development of crown roots (CRs) in rice. WOX11, a WUSCHEL-related homeobox transcription factor, is required in crown root development (Zhao et al., 2009). In *wox11* mutants, significant alteration is observed in the expression of many genes involved in the regulation of ROS homeostasis (Jiang et al., 2017). This suggests that ROS might be involved in crown root development controlled by WOX11. Accumulation of ethylene in rice CRs promotes the production of ROS under flooding. ROS together with other signals trigger epidermal cell death, thus promoting crown root emergence and elongation (Steffens et al., 2012). SA inhibits the expression of ROS scavenging-related genes, which increases ROS levels and promotes root meristem activity. However, decreased ROS levels in the *ABNORMAL INFLORESCENCE MERISTEM (AIM1)* mutant, which participates in SA synthesis, resulted in inhibition of rice crown root growth. Exogenous application of SA or H_2_O_2_ could partially restore root development (Xu et al., 2017). These results further support the interplay between SA, ethylene and ROS in rice crown root development. RSL4 (ROOT HAIR DEFECTIVE SIXLIKE 4) is a member of the auxin-responsive factor family. In *Arabidopsis*, auxin promotes the expression of a series of ROS-related genes by activating the expression of *RSL4*, thereby regulating the elongation of root hair cells, indicating that ROS also play an important role in root hair development (Mangano et al., 2017). These findings establish a molecular link between auxin and ROS-mediated polar root hair growth.

## ROS Participate in Plant Stress Responses

A great deal of evidence has shown that environmental factors such as heat (Zhao et al., 2018), cold (Kawarazaki et al., 2013), drought (Lee et al., 2012), Al toxicity (Wu et al., 2017), organic pollutants (OPs) (Ahammed et al., 2017) and pathogens (Kim and Hwang, 2014; Yang et al., 2017) could induce ROS generation in plant cells (Table 2). ROS, acting as signaling molecules, trigger signal transduction pathways in response to those stresses. On the other hand, ROS cause irreversible cellular damage through their strong oxidative properties, which promote alterations in plant morphological structures that enhance resistance (Wahid et al., 2007; Bose et al., 2014; Frederickson Matika and Loake, 2014). Because of the existence of many interconvertible ROS, it is very difficult to distinguish between the cytotoxic and signaling events that are induced by a particular ROS. It should be pointed out that although ROS cause cell death, it is a necessary process to confer resistance to stress. Altogether, stress-induced ROS-activating responses have to occur rapidly with the appearance of the stress and should decay when the stress disappears. Plants lacking AtFtSH4, an ATP-dependent mitochondrial protease, exhibited an intriguing phenotype of precocious cessation of growth at both the SAM and RAM when grown at elevated temperature (LD 31°C). This was associated with accumulation of internal oxidative stress and progressive mitochondrial dysfunction (Dolzblasz et al., 2016, 2018). These results reveal that maintaining mitochondrial functionality within the SAM and RAM, which depends on AtFtSH4, is vital to preserve stem cell activity and adaptation to temperature stress throughout development. Ethylene accumulation induces the expression of *RBOHH*, a member of the NADPH oxidase gene family. Knock out of *RBOHH* by CRISPR/Cas9 reduces ROS accumulation and inducible aerenchyma formation in rice roots, which is essential for rice to adapt to flooding and other oxygen-deficient conditions (Yamauchi et al., 2017). Under drought conditions, ABA prevents H_2_O_2_ accumulation through induction of CAT *OsCATB* expression and protects cells against ROS oxidative damage (Ye et al., 2011). When wheat E3 ubiquitin ligase TaPUB1 (The Plant U-Box Proteins 1) is transfected into tobacco, less accumulation of ROS and stronger antioxidant capacity are detected in the transgenic plants, thereby improving the survival rate of transgenic tobacco in drought stress (Zhang et al., 2017a). In *Arabidopsis*, APETALA2/ETHYLENE RESPONSE FACTOR (AP2/ERF) transcription factor RRTF1 (Redox Responsive Transcription Factor 1) is a component of the core redox signaling network. Its expression is rapidly and transiently stimulated by various ROS generated by biotic and abiotic signals. Elevated *RRTF1* levels in plants causes ROS accumulation, which suggests that RRTF1 amplifies ROS formation in response to stresses. Stimulation of ROS production by *RRTF1* might be important for a rapid and transient establishment of local ROS maxima to induce appropriate downstream responses (Matsuo et al., 2015). When pathogens invade, plants stimulate ROS production, which is rapidly triggered following detection of a pathogen and may synergistically activate the hypersensitive response (HR) (Delledonne et al., 2001). In plant disease resistance, ROS play a positive role and directly kill invading bacteria (Paiva and Bozza, 2014), and at the same time enhance thickening of adjacent cell walls to prevent spread of invading pathogens (Wang and Higgins, 2005). The rapid production of the ROS burst is a conserved signaling output in immunity across kingdoms. To protect against infection by fungal pathogens, plants have developed the pattern-recognition receptor (PRRs) for chitin perception, which triggers the intracellular activation of mitogen-activated protein kinase (MAPK) cascades for the rapid production of ROS (Kawasaki et al., 2017). In *Arabidopsis*, PRR-Associated Kinase BIK1 directly phosphorylates NADPH oxidase RBOHD and causes the PAMP-induced ROS burst and antibacterial immunity (Kadota et al., 2014). Additionally, environmental pollution by OPs also induces accumulation of both H_2_O_2_ and nitric oxide (NO) in root tips, resulting in increased malondialdehyde (MDA) content, an indicator of membrane lipid peroxidation, and abnormal root growth. Plant growth and stress tolerance regulator 24-epibrassinolide (EBR) induces non-enzymatic and enzymatic antioxidant defense systems in cucumber, and increases the content of antioxidants such as SOD, CAT, and GSH, that maintains the homeostasis of ROS in cells, consequently enhancing the resistance of cucumber to OPs (Ahammed et al., 2017).

**TABLE 2:** ROS involved in plant stress responses.

**Stress response**	**Relative ROS**	**Gene or phytohormone**	**Source**	**References**
Waterlogging	$O_{2}^{\cdot -}$, H_2_O_2_	Ethylene, *OsRBOHH*	Rice	Yamauchi et al.,2017
Water stress	H_2_O_2_	ABA, *OsCATB*	Rice	Ye et al.,2011
High temperature	$O_{2}^{\cdot -}$, H_2_O_2_	*OsCATB*	Rice	Zhao et al.,2018
Disease resistance	H_2_O_2_	Ethylene, *OsEIN2*	Rice	Yang et al.,2017
Cold temperature	$O_{2}^{\cdot -}$, H_2_O_2_	*AtSRC*	*Arabidopsis*	Kawarazaki et al.,2013
Plant immune	–	*AtRBOHD*	*Arabidopsis*	Kadota et al.,2014
Drought	$O_{2}^{\cdot -}$, H_2_O_2_	ABA, *AtNTL4*	*Arabidopsis*	Lee et al.,2012
Al stress	H_2_O_2_	*AtPRX64*	Tobacco	Wu et al.,2017
Organic pollutants treatment	H_2_O_2_, NO	24-Epibrassinolide	Cucumber	Ahammed et al.,2017
Microbial pathogens	H_2_O_2_	SA*, CaPAL1*	Pepper	Kim and Hwang,2014

*Some key genes involved in ROS homeostasis and their functions during plant stress response are listed in the table. “−” indicates that the specific types of ROS are not clearly specified in the literature. ABA, abscisic acid; SA, salicylic acid.*

## Interplay Between ROS and Epigenetic Modification

Epigenetic modifications, including both post-translational modifications of histone proteins and chemical modifications of DNA, often help regulate the expression of genes in specific redox pathways. Conversely, ROS have impacts on the epigenetic mechanisms of gene regulation. In mammals, histone deacetylases (HDACs) function in epigenetic regulation in connection with oxidative stress (Shimazu et al., 2013). HDACs can change conformation, consequently diminishing their catalytic activity or altering their cellular localization under oxidative stress (Doyle and Fitzpatrick, 2010). On the other hand, increases in ROS result in increases in various histone modifications such as H3K4me2/3, H3K79me3, H3k27me3, and H3K9me2, due to inhibition of histone demethylases (Chen et al., 2006; Zhou et al., 2008; Niu et al., 2015). Growing evidence reveals a close link between ROS metabolism and epigenetic regulation during plant growth and environmental acclimation. Four distinct DNA demethylases, REPRESSOR OF SILENCING 1 (ROS1), DEMETER (DME), DME-like 2 (DML2), and DML3 catalyzed the active removal of 5-methylcytosine from DNA (Zhang and Zhu, 2012; Wang et al., 2016). Recent studies demonstrate that ROS1 and DME interact directly with the Fe–S cluster assembly machinery, which is highly susceptible to oxidation by ROS. Their activity can consequently be altered by stress-derived oxidative conditions (Shen et al., 2016). This result reveals a connection between DNA methylation and ROS metabolism. Rice plants overexpressing *OsSRT1*, a SILENT INFORMATION REGULATOR2 (SIR2)-related HDAC gene, have shown an enhanced tolerance to oxidative stress, while *OsSRT1* RNAi induces H_2_O_2_ overproduction, DNA fragmentation, and cell death (Huang et al., 2007). Recent studies also showed that OsSRT1 not only inhibits the “moonlighting” transcriptional activation activity of glyceraldehyde-3-phosphatedehydrogenase (GAPDH),which binds the promoters of glycolytic genes and stimulates their expression, but also reduces the acetylation of GAPDH lysine residues and its nuclear accumulation that are otherwise enhanced by oxidative stress in rice seedlings (Zhang et al., 2017b). Cellular oxidation could reduce HDA19 and HDA9 activity, thereby enhancing histone acetylation and transcription of stress-responsive genes in *Arabidopsis* (Liu et al., 2015). Recent studies also showed that changes in ROS levels caused apparent epigenetic modifications such as acylation, which in turn regulated the activity of ROS related proteins in rice leaves (Zhou et al., 2018). This suggests that the interplay between ROS and acylation might play important roles in the PTMs (post-translational modifications) of leaf proteins that have key metabolic functions. We also found that the expression of some ROS-related genes was linked to changes in their acetylation modification during crown root development in rice (Jiang et al., 2017). This implies there is close cooperation between ROS and epigenetic regulation of gene expression during rice crown root development. However, further studies are needed regarding how interactions between ROS and epigenetic modifications regulate gene expression. Histone demethylation is catalyzed by two different classes of enzymes: the jumonji C (JmjC) demethylases, which are Fe (II)- and 2-oxoglutarate-dependent dioxygenases, and FAD-dependent amino oxidases, including lysine-specific demethylase 1 (LSD1) (Chen et al., 2011). There is a clear connection between histone methylation, energy metabolism, and cell redox balance in animals and yeast cells (Niu et al., 2015). Many JmjC proteins have been reported to respond to plant exposure to stress by modulating the expression of stress-related genes, probably acting together with ROS generated during stressful conditions, leading to the establishment of a complex network of defense responses (Shen et al., 2016). However, it is not clear whether the activity of JmjC proteins is directly altered by ROS. In mammalian cells, glutathione (GSH) seems to be a new post-translational modifier of the histone code, capable of modulating the chromatin structure. Glutathionylation of histone H3 affects nucleosome stability leading to a more open chromatin structure (Garcia-Gimenez and Pallardo, 2014). This reveals a new connection between epigenetic control and cellular redox homeostasis. In addition, GSH can influence epigenetic processes, inhibiting the activity of the enzymes involved in the synthesis of *S*-adenosyl-methionine (SAM), which is used by DNA methyltransferases (DNMTs) and HMTs as a substrate for DNA and histone methylation, respectively (Garcia-Gimenez and Pallardo, 2014; Garcia-Gimenez et al., 2017). Therefore, the modulation of GSH metabolism may control oxidative stress and epigenetic mechanisms. However, this still needs to be extensively investigated in plants.

## Discussion and Prospects

During recent years, sources of ROS, mechanisms of production and removal, and key antioxidant molecules and enzymes that scavenge ROS have been reported in plants. However, much of our current knowledge about ROS remains unclear. Firstly, most ROS have short half-lives and are prone to chemical reactions to produce water or secondary ROS. It is still difficult to accurately study how various ROS lead to signaling and drive plant growth and development in a strictly localized and timely manner. Secondly, we do not understand the interplay between the spatio-temporal production of various ROS and their activities. In certain cases, it is very difficult to distinguish whether oxidative stress is the cause or an effect of cellular damage. This restricts our further understanding of their roles in plants. Thirdly, the abnormal accumulation of ROS also leads to oxidative modification of some microRNA and proteins. The mismatch between oxidized miRNAs and proteins might be involved in the initiation of apoptosis, that eventually leads to the cell death (Wang et al., 2015; Dumont and Rivoal, 2019; Shekhova et al., 2019; Smirnoff and Arnaud, 2019). These findings shed new light on our current understanding of the significance of ROS functions. It will be interesting to characterize whether oxidatively modified miRNA or proteins are involved in plant growth and development. Finally, recent studies showed that interplay between ROS levels and epigenetic modifications had important roles in plant development and stress responses, and biotic and abiotic stresses greatly affected plant growth and redox states. However, the regulatory mechanism remains unknown. It will be important to explore the cross-talk between ROS and epigenetic modifications, which will contribute to understanding the mechanisms whereby ROS homeostasis, epigenetics and plant adaptation and tolerance are mutually regulated.

## Author Contributions

YZ wrote and revised the manuscript. HH collected all the materials and wrote the draft. FU and D-XZ revised the manuscript. MY gave some suggestion and advice.

## Conflict of Interest Statement

The authors declare that the research was conducted in the absence of any commercial or financial relationships that could be construed as a potential conflict of interest.

## References

[B1] Ahammed G. J. He B. B. Qian X. J. Zhou Y. H. Shi K. Zhou J. (2017). 24-Epibrassinolide alleviates organic pollutants-retarded root elongation by promoting redox homeostasis and secondary metabolism in *Cucumis sativus* L. *Environ. Pollut.* 229 922–931. 10.1016/j.envpol.2017.07.076 28774551

[B2] Apel K. Hirt H. (2004). Reactive oxygen species: metabolism, oxidative stress, and signal transduction. *Annu. Rev. Plant Biol.* 55 373–399. 10.1146/annurev.arplant.55.031903.141701 15377225

[B3] Baek D. Cha J. Y. Kang S. Park B. Lee H. J. Hong H. (2015). The *Arabidopsis* a zinc finger domain protein ARS1 is essential for seed germination and ROS homeostasis in response to ABA and oxidative stress. *Front. Plant Sci.* 6:963 10.3389/fpls.2015.00963 PMC4631831 26583028

[B4] Bienert G. P. Moller A. L. Kristiansen K. A. Schulz A. Moller I. M. Schjoerring J. K. (2007). Specific aquaporins facilitate the diffusion of hydrogen peroxide across membranes. *J. Biol. Chem.* 282 1183–1192. 10.1074/jbc.M603761200 17105724

[B5] Blokhina O. (2003). Antioxidants, oxidative damage and oxygen deprivation stress: a review. *Ann. Bot.* 91 179–194. 10.1093/aob/mcf118 12509339 PMC4244988

[B6] Bose J. Rodrigo-Moreno A. Shabala S. (2014). ROS homeostasis in halophytes in the context of salinity stress tolerance. *J. Exp. Bot.* 65 1241–1257. 10.1093/jxb/ert430 24368505

[B7] Chen H. Ke Q. Kluz T. Yan Y. Costa M. (2006). Nickel ions increase histone H3 lysine 9 dimethylation and induce transgene silencing. *Mol. Cell. Biol.* 26 3728–3737. 10.1128/MCB.26.10.3728-3737.2006 16648469 PMC1488989

[B8] Chen X. Hu Y. Zhou D. X. (2011). Epigenetic gene regulation by plant Jumonji group of histone demethylase. *Biochim. Biophys. Acta* 1809 421–426. 10.1016/j.bbagrm.2011.03.004 21419882

[B9] de Simone A. Hubbard R. de la Torre N. V. Velappan Y. Wilson M. Considine M. J. (2017). Redox changes during the cell cycle in the embryonic root meristem of *Arabidopsis thaliana*. *Antioxid. Redox Signal.* 27 1505–1519. 10.1089/ars.2016.6959 28457165 PMC5678362

[B10] Delledonne M. Zeier J. Marocco A. Lamb C. (2001). Signal interactions between nitric oxide and reactive oxygen intermediates in the plant hypersensitive disease resistance response. *Proc. Natl. Acad. Sci. U.S.A.* 98 13454–13459. 10.1073/pnas.231178298 11606758 PMC60892

[B11] Dietz K. J. Turkan I. Krieger-Liszkay A. (2016). Redox- and reactive oxygen species-dependent signaling into and out of the photosynthesizing chloroplast. *Plant Physiol.* 171 1541–1550. 10.1104/pp.16.00375 27255485 PMC4936569

[B12] Dolzblasz A. Gola E. M. Sokolowska K. Smakowska-Luzan E. Twardawska A. Janska H. (2018). Impairment of meristem proliferation in plants lacking the mitochondrial protease AtFTSH4. *Int. J. Mol. Sci.* 19:853 10.3390/ijms19030853 PMC5877714 29538317

[B13] Dolzblasz A. Smakowska E. Gola E. M. Sokolowska K. Kicia M. Janska H. (2016). The mitochondrial protease AtFTSH4 safeguards *Arabidopsis* shoot apical meristem function. *Sci. Rep.* 6:28315 10.1038/srep28315 PMC4913265 27321362

[B14] Doyle K. Fitzpatrick F. A. (2010). Redox signaling, alkylation (carbonylation) of conserved cysteines inactivates class I histone deacetylases 1, 2, and 3 and antagonizes their transcriptional repressor function. *J. Biol. Chem.* 285 17417–17424. 10.1074/jbc.M109.089250 20385560 PMC2878505

[B15] Dumont S. Rivoal J. (2019). Consequences of oxidative stress on plant glycolytic and respiratory metabolism. *Front. Plant Sci.* 10:166 10.3389/fpls.2019.00166 PMC6387960 30833954

[B16] Dunand C. Crevecoeur M. Penel C. (2007). Distribution of superoxide and hydrogen peroxide in *Arabidopsis* root and their influence on root development: possible interaction with peroxidases. *New Phytol.* 174 332–341. 10.1111/j.1469-8137.2007.01995.x 17388896

[B17] Foyer C. H. Noctor G. (2016). Stress-triggered redox signalling: what’s in pROSpect? *Plant Cell Environ.* 39 951–964. 10.1111/pce.12621 26264148

[B18] Frederickson Matika D. E. Loake G. J. (2014). Redox regulation in plant immune function. *Antioxid. Redox Signal.* 21 1373–1388. 10.1089/ars.2013.5679 24206122 PMC4158969

[B19] Fujita M. Fujita Y. Noutoshi Y. Takahashi F. Narusaka Y. Yamaguchi-Shinozaki K. (2006). Crosstalk between abiotic and biotic stress responses: a current view from the points of convergence in the stress signaling networks. *Curr. Opin. Plant Biol.* 9 436–442. 10.1016/j.pbi.2006.05.014 16759898

[B20] Garcia-Gimenez J. L. Pallardo F. V. (2014). Maintenance of glutathione levels and its importance in epigenetic regulation. *Front. Pharmacol.* 5:88 10.3389/fphar.2014.00088 PMC4017153 24847264

[B21] Garcia-Gimenez J. L. Roma-Mateo C. Perez-Machado G. Peiro-Chova L. Pallardo F. V. (2017). Role of glutathione in the regulation of epigenetic mechanisms in disease. *Free Radic. Biol. Med.* 112 36–48. 10.1016/j.freeradbiomed.2017.07.008 28705657

[B22] Gechev T. S. Van Breusegem F. Stone J. M. Denev I. Laloi C. (2006). Reactive oxygen species as signals that modulate plant stress responses and programmed cell death. *Bioessays* 28 1091–1101. 10.1002/bies.20493 17041898

[B23] Gill S. S. Tuteja N. (2010). Reactive oxygen species and antioxidant machinery in abiotic stress tolerance in crop plants. *Plant Physiol. Biochem.* 48 909–930. 10.1016/j.plaphy.2010.08.016 20870416

[B24] Hiramoto K. Ojima N. Sako K. Kikugawa K. (1996). Effect of plant phenolics on the formation of the spin-adduct of hydroxyl radical and the DNA strand breaking by hydroxyl radical. *Biol. Pharm. Bull.* 19 558–563. 10.1248/bpb.19.558 8860958

[B25] Hu L. Liang W. Yin C. Cui X. Zong J. Wang X. (2011). Rice MADS3 regulates ROS homeostasis during late anther development. *Plant Cell* 23 515–533. 10.1105/tpc.110.074369 21297036 PMC3077785

[B26] Huang L. Jia J. Zhao X. Zhang M. Huang X. Ji E. (2018). The ascorbate peroxidase APX1 is a direct target of a zinc finger transcription factor ZFP36 and a late embryogenesis abundant protein OsLEA5 interacts with ZFP36 to co-regulate OsAPX1 in seed germination in rice. *Biochem. Biophys. Res. Commun.* 495 339–345. 10.1016/j.bbrc.2017.10.128 29106954

[B27] Huang L. Sun Q. Qin F. Li C. Zhao Y. Zhou D. X. (2007). Down-regulation of a SILENT INFORMATION REGULATOR2-related histone deacetylase gene, OsSRT1, induces DNA fragmentation and cell death in rice. *Plant Physiol.* 144 1508–1519. 10.1104/pp.107.099473 17468215 PMC1914135

[B28] Huang L. Yu L. J. Zhang X. Fan B. Wang F. Z. Dai Y. S. (2019). Autophagy regulates glucose-mediated root meristem activity by modulating ROS production in *Arabidopsis*. *Autophagy* 15 407–422. 10.1080/15548627.2018.1520547 30208757 PMC6351127

[B29] Huang S. Van Aken O. Schwarzlander M. Belt K. Millar A. H. (2016). The roles of mitochondrial reactive oxygen species in cellular signaling and stress response in plants. *Plant Physiol.* 171 1551–1559. 10.1104/pp.16.00166 27021189 PMC4936549

[B30] Ishibashi Y. Kasa S. Sakamoto M. Aoki N. Kai K. Yuasa T. (2015). A role for reactive oxygen species produced by NADPH oxidases in the embryo and aleurone cells in barley seed germination. *PLoS One* 10:e0143173 10.1371/journal.pone.0143173 PMC4651353 26579718

[B31] Jacobowitz J. R. Doyle W. C. Weng J. K. (2019). PRX9 and PRX40 are extensin peroxidases essential for maintaining tapetum and microspore cell wall integrity during *Arabidopsis* anther development. *Plant Cell* 31 848–861. 10.1105/tpc.18.00907 30886127 PMC6501601

[B32] Jiang W. Zhou S. Zhang Q. Song H. Zhou D. X. Zhao Y. (2017). Transcriptional regulatory network of WOX11 is involved in the control of crown root development, cytokinin signals, and redox in rice. *J. Exp. Bot.* 68 2787–2798. 10.1093/jxb/erx153 28830102 PMC5853245

[B33] Kadota Y. Sklenar J. Derbyshire P. Stransfeld L. Asai S. Ntoukakis V. (2014). Direct regulation of the NADPH oxidase RBOHD by the PRR-associated kinase BIK1 during plant immunity. *Mol. Cell* 54 43–55. 10.1016/j.molcel.2014.02.021 24630626

[B34] Karkonen A. Kuchitsu K. (2015). Reactive oxygen species in cell wall metabolism and development in plants. *Phytochemistry* 112 22–32. 10.1016/j.phytochem.2014.09.016 25446232

[B35] Kawarazaki T. Kimura S. Iizuka A. Hanamata S. Nibori H. Michikawa M. (2013). A low temperature-inducible protein AtSRC2 enhances the ROS-producing activity of NADPH oxidase AtRbohF. *Biochim. Biophys. Acta* 1833 2775–2780. 10.1016/j.bbamcr.2013.06.024 23872431

[B36] Kawasaki T. Yamada K. Yoshimura S. Yamaguchi K. (2017). Chitin receptor-mediated activation of MAP kinases and ROS production in rice and *Arabidopsis*. *Plant Signal. Behav.* 12:e1361076 10.1080/15592324.2017.1361076 PMC5640189 28805500

[B37] Kim D. S. Hwang B. K. (2014). An important role of the pepper phenylalanine ammonia-lyase gene (PAL1) in salicylic acid-dependent signalling of the defence response to microbial pathogens. *J. Exp. Bot.* 65 2295–2306. 10.1093/jxb/eru109 24642849 PMC4036500

[B38] Kka N. Rookes J. Cahill D. (2018). The influence of ascorbic acid on root growth and the root apical meristem in Arabidopsis thaliana. *Plant Physiol. Biochem.* 129 323–330. 10.1016/j.plaphy.2018.05.031 29929127

[B39] Kong X. Tian H. Yu Q. Zhang F. Wang R. Gao S. (2018). PHB3 maintains root stem cell niche identity through ROS-Responsive AP2/ERF transcription factors in *Arabidopsis*. *Cell Rep.* 22 1350–1363. 10.1016/j.celrep.2017.12.105 29386120

[B40] Lee S. Seo P. J. Lee H.-J. Park C.-M. (2012). A NAC transcription factor NTL4 promotes reactive oxygen species production during drought-induced leaf senescence in *Arabidopsis*. *Plant J.* 70 831–844. 10.1111/j.1365-313X.2012.04932.x 22313226

[B41] Liu B. Zhao S. Tan F. Zhao H. Wang D. Si H. (2017). Changes in ROS production and antioxidant capacity during tuber sprouting in potato. *Food Chem.* 237 205–213. 10.1016/j.foodchem.2017.05.107 28763987

[B42] Liu P. Zhang H. Yu B. Xiong L. Xia Y. (2015). Proteomic identification of early salicylate- and flg22-responsive redox-sensitive proteins in *Arabidopsis*. *Sci. Rep.* 5:8625 10.1038/srep08625 PMC4342551 25720653

[B43] Lv B. Tian H. Zhang F. Liu J. Lu S. Bai M. (2018). Brassinosteroids regulate root growth by controlling reactive oxygen species homeostasis and dual effect on ethylene synthesis in *Arabidopsis*. *PLoS Genet.* 14:e1007144 10.1371/journal.pgen.1007144 PMC5783399 29324765

[B44] Mangano S. Denita-Juarez S. P. Choi H. S. Marzol E. Hwang Y. Ranocha P. (2017). Molecular link between auxin and ROS-mediated polar growth. *Proc. Natl. Acad. Sci. U.S.A.* 114 5289–5294. 10.1073/pnas.1701536114 28461488 PMC5441824

[B45] Manzano C. Pallero-Baena M. Casimiro I. De Rybel B. Orman-Ligeza B. Van Isterdael G. (2014). The emerging role of reactive oxygen species signaling during lateral root development. *Plant Physiol.* 165 1105–1119. 10.1104/pp.114.238873 24879433 PMC4081325

[B46] Matsuo M. Johnson J. M. Hieno A. Tokizawa M. Nomoto M. Tada Y. (2015). High redox responsive transcription factor1 levels result in accumulation of reactive oxygen species in *Arabidopsis thaliana* shoots and roots. *Mol. Plant* 8 1253–1273. 10.1016/j.molp.2015.03.011 25882345

[B47] Mhamdi A. Van Breusegem F. (2018). Reactive oxygen species in plant development. *Development* 145:dev164376 10.1242/dev.164376 30093413

[B48] Miller E. W. Dickinson B. C. Chang C. J. (2010). Aquaporin-3 mediates hydrogen peroxide uptake to regulate downstream intracellular signaling. *Proc. Natl. Acad. Sci. U.S.A.* 107 15681–15686. 10.1073/pnas.1005776107 20724658 PMC2936599

[B49] Miller G. Suzuki N. Ciftci-Yilmaz S. Mittler R. (2010). Reactive oxygen species homeostasis and signalling during drought and salinity stresses. *Plant Cell Environ.* 33 453–467. 10.1111/j.1365-3040.2009.02041.x 19712065

[B50] Mittler R. (2017). ROS are good. *Trends Plant Sci.* 22 11–19. 10.1016/j.tplants.2016.08.002 27666517

[B51] Mittler R. Vanderauwera S. Gollery M. Van Breusegem F. (2004). Reactive oxygen gene network of plants. *Trends Plant Sci.* 9 490–498. 10.1016/j.tplants.2004.08.009 15465684

[B52] Moller I. M. Jensen P. E. Hansson A. (2007). Oxidative modifications to cellular components in plants. *Annu. Rev. Plant Biol.* 58 459–481. 10.1146/annurev.arplant.58.032806.103946 17288534

[B53] Niu Y. DesMarais T. L. Tong Z. Yao Y. Costa M. (2015). Oxidative stress alters global histone modification and DNA methylation. *Free Radic. Biol. Med.* 82 22–28. 10.1016/j.freeradbiomed.2015.01.028 25656994 PMC4464695

[B54] Paiva C. N. Bozza M. T. (2014). Are reactive oxygen species always detrimental to pathogens? *Antioxid. Redox Signal.* 20 1000–1037. 10.1089/ars.2013.5447 23992156 PMC3924804

[B55] Para A. Muhammad D. Orozco-Nunnelly D. A. Memishi R. Alvarez S. Naldrett M. J. (2016). The dehydratase ADT3 affects ROS homeostasis and cotyledon development. *Plant Physiol.* 172 1045–1060. 10.1104/pp.16.00464 27540109 PMC5047074

[B56] Peng Y. Hou F. Bai Q. Xu P. Liao Y. Zhang H. (2018). Rice calcineurin B-like protein-interacting protein kinase 31 (OsCIPK31) is involved in the development of panicle apical spikelets. *Front. Plant Sci.* 9:1661 10.3389/fpls.2018.01661 PMC6262370 30524455

[B57] Ribeiro C. W. Korbes A. P. Garighan J. A. Jardim-Messeder D. Carvalho F. E. L. Sousa R. H. V. (2017). Rice peroxisomal ascorbate peroxidase knockdown affects ROS signaling and triggers early leaf senescence. *Plant Sci.* 263 55–65. 10.1016/j.plantsci.2017.07.009 28818384

[B58] Rosing M. T. Frei R. (2004). U-rich archaean sea-floor sediments from Greenland – Indications of >3700 Ma oxygenic photosynthesis. *Earth Planet. Sci. Lett.* 217 237–244. 10.1016/s0012-821x(03)00609-5

[B59] Sandalio L. M. Romero-Puertas M. C. (2015). Peroxisomes sense and respond to environmental cues by regulating ROS and RNS signalling networks. *Ann. Bot.* 116 475–485. 10.1093/aob/mcv074 26070643 PMC4577995

[B60] Schippers J. H. Foyer C. H. van Dongen J. T. (2016). Redox regulation in shoot growth, SAM maintenance and flowering. *Curr. Opin. Plant Biol.* 29 121–128. 10.1016/j.pbi.2015.11.009 26799134

[B61] Schippers J. H. Nguyen H. M. Lu D. Schmidt R. Mueller-Roeber B. (2012). ROS homeostasis during development: an evolutionary conserved strategy. *Cell. Mol. Life Sci.* 69 3245–3257. 10.1007/s00018-012-1092-4 22842779 PMC11114851

[B62] Shekhova E. Ivanova L. Kruger T. Stroe M. C. Macheleidt J. Kniemeyer O. (2019). Redox proteomic analysis reveals oxidative modifications of proteins by increased levels of intracellular reactive oxygen species during hypoxia adaptation of aspergillus fumigatus. *Proteomics* 19:e1800339 10.1002/pmic.201800339 30632700

[B63] Shen Y. Issakidis-Bourguet E. Zhou D. X. (2016). Perspectives on the interactions between metabolism, redox, and epigenetics in plants. *J. Exp. Bot.* 67 5291–5300. 10.1093/jxb/erw310 27531885

[B64] Shimazu T. Hirschey M. D. Newman J. He W. Shirakawa K. Le Moan N. (2013). Suppression of oxidative stress by beta-hydroxybutyrate, an endogenous histone deacetylase inhibitor. *Science* 339 211–214. 10.1126/science.1227166 23223453 PMC3735349

[B65] Smirnoff N. Arnaud D. (2019). Hydrogen peroxide metabolism and functions in plants. *New Phytol.* 221 1197–1214. 10.1111/nph.15488 30222198

[B66] Steffens B. Kovalev A. Gorb S. N. Sauter M. (2012). Emerging roots alter epidermal cell fate through mechanical and reactive oxygen species signaling. *Plant Cell* 24 3296–3306. 10.1105/tpc.112.101790 22904148 PMC3462632

[B67] Tian Y. Fan M. Qin Z. Lv H. Wang M. Zhang Z. (2018). Hydrogen peroxide positively regulates brassinosteroid signaling through oxidation of the BRASSINAZOLE-RESISTANT1 transcription factor. *Nat. Commun.* 9:1063 10.1038/s41467-018-03463-x PMC5852159 29540799

[B68] Tognetti V. B. Bielach A. Hrtyan M. (2017). Redox regulation at the site of primary growth: auxin, cytokinin and ROS crosstalk. *Plant Cell Environ.* 40 2586–2605. 10.1111/pce.13021 28708264

[B69] Tsukagoshi H. Busch W. Benfey P. N. (2010). Transcriptional regulation of ROS controls transition from proliferation to differentiation in the root. *Cell* 143 606–616. 10.1016/j.cell.2010.10.020 21074051

[B70] Viola I. L. Guttlein L. N. Gonzalez D. H. (2013). Redox modulation of plant developmental regulators from the class I TCP transcription factor family. *Plant Physiol.* 162 1434–1447. 10.1104/pp.113.216416 23686421 PMC3707549

[B71] Wahid A. Gelani S. Ashraf M. Foolad M. (2007). Heat tolerance in plants: an overview. *Environ. Exp. Bot.* 61 199–223. 10.1016/j.envexpbot.2007.05.011

[B72] Wang J. Higgins V. J. (2005). Nitric oxide modulates H2O2-mediated defenses in the Colletotrichum coccodes-tomato interaction. *Physiol. Mol. Plant Pathol.* 67 131–137. 10.1016/j.pmpp.2005.11.002

[B73] Wang J. X. Gao J. Ding S. L. Wang K. Jiao J. Q. Wang Y. (2015). Oxidative modification of miR-184 enables it to target Bcl-xL and Bcl-w. *Mol. Cell* 59 50–61. 10.1016/j.molcel.2015.05.003 26028536

[B74] Wang X. Li Q. Yuan W. Cao Z. Qi B. Kumar S. (2016). The cytosolic Fe-S cluster assembly component MET18 is required for the full enzymatic activity of ROS1 in active DNA demethylation. *Sci. Rep.* 6:26443 10.1038/srep26443 PMC4872223 27193999

[B75] Waszczak C. Kerchev P. I. Muhlenbock P. Hoeberichts F. A. Van Der Kelen K. Mhamdi A. (2016). SHORT-ROOT deficiency alleviates the cell death phenotype of the *Arabidopsis* catalase2 mutant under photorespiration-promoting conditions. *Plant Cell* 28 1844–1859. 10.1105/tpc.16.00038 27432873 PMC5006698

[B76] Wu Y. Yang Z. How J. Xu H. Chen L. Li K. (2017). Overexpression of a peroxidase gene (AtPrx64) of Arabidopsis thaliana in tobacco improves plant’s tolerance to aluminum stress. *Plant Mol. Biol.* 95 157–168. 10.1007/s11103-017-0644-2 28815457

[B77] Wudick M. M. Li X. J. Valentini V. Geldner N. Chory J. Lin J. X. (2015). Subcellular redistribution of root aquaporins induced by hydrogen peroxide. *Mol. Plant* 8 1103–1114. 10.1016/j.molp.2015.02.017 25749111

[B78] Xie H. T. Wan Z. Y. Li S. Zhang Y. (2014). Spatiotemporal production of reactive oxygen species by NADPH oxidase is critical for tapetal programmed cell death and pollen development in Arabidopsis. *Plant Cell* 26 2007–2023. 10.1105/tpc.114.125427 24808050 PMC4079365

[B79] Xu L. Zhao H. Ruan W. Deng M. Wang F. Peng J. (2017). ABNORMAL INFLORESCENCE MERISTEM1 functions in salicylic acid biosynthesis to maintain proper reactive oxygen species levels for root meristem activity in Rice. *Plant Cell* 29 560–574. 10.1105/tpc.16.00665 28298519 PMC5385951

[B80] Yamauchi T. Yoshioka M. Fukazawa A. Mori H. Nishizawa N. K. Tsutsumi N. (2017). An NADPH Oxidase RBOH functions in rice roots during lysigenous aerenchyma formation under oxygen-deficient conditions. *Plant Cell* 29 775–790. 10.1105/tpc.16.00976 28351990 PMC5435434

[B81] Yang C. Li W. Cao J. Meng F. Yu Y. Huang J. (2017). Activation of ethylene signaling pathways enhances disease resistance by regulating ROS and phytoalexin production in rice. *Plant J.* 89 338–353. 10.1111/tpj.13388 27701783

[B82] Ye N. Zhu G. Liu Y. Li Y. Zhang J. (2011). ABA controls H2O2 accumulation through the induction of OsCATB in rice leaves under water stress. *Plant Cell Physiol.* 52 689–698. 10.1093/pcp/pcr028 21398647

[B83] Yu Q. Tian H. Yue K. Liu J. Zhang B. Li X. (2016). A P-Loop NTPase regulates quiescent center cell division and distal stem cell identity through the regulation of ROS homeostasis in *Arabidopsis* root. *PLoS Genet.* 12:e1006175 10.1371/journal.pgen.1006175 PMC5008728 27583367

[B84] Yu X. Pasternak T. Eiblmeier M. Ditengou F. Kochersperger P. Sun J. (2013). Plastid-localized glutathione reductase2-regulated glutathione redox status is essential for *Arabidopsis* root apical meristem maintenance. *Plant Cell* 25 4451–4468. 10.1105/tpc.113.117028 24249834 PMC3875729

[B85] Zafra A. Rodriguez-Garcia M. I. Alche Jde D. (2010). Cellular localization of ROS and NO in olive reproductive tissues during flower development. *BMC Plant Biol.* 10:36 10.1186/1471-2229-10-36 PMC2838403 20181244

[B86] Zeng J. Dong Z. Wu H. Tian Z. Zhao Z. (2017). Redox regulation of plant stem cell fate. *EMBO J.* 36 2844–2855. 10.15252/embj.201695955 28838936 PMC5623875

[B87] Zhang G. Zhang M. Zhao Z. Ren Y. Li Q. Wang W. (2017a). Wheat TaPUB1 modulates plant drought stress resistance by improving antioxidant capability. *Sci. Rep.* 7:7549 10.1038/s41598-017-08181-w PMC5548723 28790447

[B88] Zhang H. Zhao Y. Zhou D. X. (2017b). Rice NAD+-dependent histone deacetylase OsSRT1 represses glycolysis and regulates the moonlighting function of GAPDH as a transcriptional activator of glycolytic genes. *Nucleic Acids Res.* 45 12241–12255. 10.1093/nar/gkx825 28981755 PMC5716216

[B89] Zhang S. Li C. Wang R. Chen Y. Shu S. Huang R. (2017c). The *Arabidopsis* mitochondrial protease FtSH4 is involved in leaf senescence via regulation of WRKY-dependent salicylic acid accumulation and signaling. *Plant Physiol.* 173 2294–2307. 10.1104/pp.16.00008 28250067 PMC5373041

[B90] Zhang H. Zhang T. T. Liu H. Shi Y. Wang M. Bie X. M. (2018). Thioredoxin-mediated ROS homeostasis explains natural variation in plant regeneration. *Plant Physiol.* 176 2231–2250. 10.1104/pp.17.00633 28724620 PMC5841725

[B91] Zhang H. Zhu J. K. (2012). Active DNA demethylation in plants and animals. *Cold Spring Harb. Symp. Quant. Biol.* 77 161–173. 10.1101/sqb.2012.77.014936 23197304 PMC3657592

[B92] Zhao Q. Zhou L. Liu J. Cao Z. Du X. Huang F. (2018). Involvement of CAT in the detoxification of HT-induced ROS burst in rice anther and its relation to pollen fertility. *Plant Cell Rep.* 37 741–757. 10.1007/s00299-018-2264-y 29464319

[B93] Zhao Y. Hu Y. Dai M. Huang L. Zhou D. X. (2009). The WUSCHEL-related homeobox gene WOX11 is required to activate shoot-borne crown root development in rice. *Plant Cell* 21 736–748. 10.1105/tpc.108.061655 19258439 PMC2671696

[B94] Zhou H. Finkemeier I. Guan W. Tossounian M. A. Wei B. Young D. (2018). Oxidative stress-triggered interactions between the succinyl- and acetyl-proteomes of rice leaves. *Plant Cell Environ.* 41 1139–1153. 10.1111/pce.13100 29126343

[B95] Zhou X. Sun H. Ellen T. P. Chen H. Costa M. (2008). Arsenite alters global histone H3 methylation. *Carcinogenesis* 29 1831–1836. 10.1093/carcin/bgn063 18321869 PMC2722848

[B96] Zimmermann P. Heinlein C. Orendi G. Zentgraf U. (2006). Senescence-specific regulation of catalases in *Arabidopsis thaliana* (L.) Heynh. *Plant Cell Environ.* 29 1049–1060. 10.1111/j.1365-3040.2005.01459.x 17080932

